# Suicidal behaviour and fear of deportation among refugees - possibilities and limitations of mental health care systems: data from a German stepped care project

**DOI:** 10.1192/j.eurpsy.2026.10367

**Published:** 2026-07-31

**Authors:** V. Mohwinkel

**Affiliations:** Clinic for Psychiatry and Psychotherapy I, Ulm University, Ravensburg, Germany

## Abstract

**Abstract:**

Recent studies indicate an increased risk of suicide among refugees compared to the native population. In this context, scientific research is focusing on the significance of postmigrative stress factors. One factor that is receiving particular attention is the residence status. The correlation between an uncertain residence status and increased psychological distress has been shown by many studies. However, the extent to which fear of deportation influences the suicidal behaviour of refugees has received comparatively little attention, so far. This presentation provides an overview of current research, completed by data from the stepped-care project refuKey (founded by NTFN Psychosoziale Zentren gGmbH and the German Society for Psychiatry and Psychotherapy, Psychosomatics and Neurology). RefuKey aims to improve the needs-based care of mentally distressed refugees in Germany. Four-fifths of the refuKey patients live with an uncertain residence status. Also, many patients report suicidal ideations at the beginning of treatment. Although the level of postmigrative stress that is experienced by refuKey patients stays high, the amount of suicidal ideations decrease significantly during the treatment. Finally, the possibilities and limitations of treating suicidal refugees who are in fear of deportation will be discussed.

**Disclosure of Interest:**

None Declared

